# Moyamoya Disease Presenting as a Transient Ischemic Attack Following Recurrent Intracerebral and Intraventricular Hemorrhage

**DOI:** 10.7759/cureus.106952

**Published:** 2026-04-13

**Authors:** Natia Babukhadia, Tornike Jangirashvili, Lolita Shengelia

**Affiliations:** 1 Neurology, Tbilisi Central Hospital, Tbilisi, GEO; 2 Neurology, Georgian American University, Tbilisi, GEO; 3 Research, Georgian American University, Tbilisi, GEO

**Keywords:** cerebrovascular disease (cvd), collateral circulation, ct angiography, intracranial hemorrhage (ich), moyamoya disease (mmd), puff of smoke appearance, transient ischemic attack

## Abstract

Moyamoya disease (MMD) is a rare, progressive cerebrovascular disorder characterized by stenosis of the internal carotid arteries and their branches, with compensatory development of abnormal, fragile collateral vessels at the base of the brain. It has a bimodal age distribution, is more prevalent in females, and typically manifests as cerebral ischemia or hemorrhage with a progressive clinical course. The co-occurrence of recurrent hemorrhagic events followed by transient ischemic episodes within the same disease course represents a particularly uncommon and diagnostically challenging clinical presentation. We report a 45-year-old female with a prior history of intracerebral hemorrhage with intraventricular extension, who recovered without residual neurological deficits. The patient subsequently presented to the Emergency Department via Emergency Medical Service with a several-day history of severe headache, dizziness, and arterial hypertension, with symptom intensification in the hours preceding admission. Notably, the patient reported transient weakness of the right upper limb accompanied by brief speech disturbance prior to hospital arrival, which resolved completely within 24 hours. On arrival, blood pressure was 154/90 mmHg, and neurological examination revealed a Glasgow Coma Scale (GCS) of 15 (E4V5M6), intact orientation and speech, full motor strength in all four extremities, symmetric reflexes, no pathological signs, and no meningeal irritation, consistent with a transient ischemic attack (TIA) without persistent neurological deficit. Cerebral CT angiography excluded acute intracranial pathology but identified a markedly atypical vascular pattern: reduced trunk-type contrast filling in the anterior circulation with compensatory collateral hemodynamic circulation, highly suggestive of MMD. Digital subtraction angiography (DSA) and neurosurgical consultation were recommended for definitive vascular characterization. The patient was managed with symptomatic treatment and dynamic monitoring, demonstrated clinical improvement with hemodynamic stabilization, and was discharged with appropriate recommendations. This case underscores the complex and heterogeneous clinical spectrum of MMD, in which a patient may experience recurrent hemorrhagic events followed by ischemic manifestations such as TIA within the same disease course. The diagnosis was established non-invasively through CT angiography, which revealed a characteristic collateral vascular pattern in the absence of acute pathology. Clinicians should maintain a high index of suspicion for MMD in patients presenting with mixed cerebrovascular events, as early recognition and prompt referral for advanced vascular imaging and neurosurgical evaluation are critical for optimizing long-term outcomes.

## Introduction

Moyamoya disease (MMD) is a rare, chronic, and progressive cerebrovascular disorder characterized by bilateral stenosis or occlusion of the terminal internal carotid arteries and their proximal branches, with subsequent development of abnormal collateral vascular networks at the base of the brain [1]. The term "moyamoya" is derived from the Japanese word meaning "puff of smoke," reflecting the hazy angiographic appearance of these collateral vessels on cerebral angiography [2]. First described in Japan in the 1960s, the disease was initially considered exclusive to East Asian populations; however, increasing global case recognition has since demonstrated its occurrence across diverse ethnic groups and geographic regions, underscoring the importance of maintaining clinical awareness beyond traditionally high-prevalence populations [3].

The underlying pathophysiology involves progressive narrowing of the supraclinoid portion of the internal carotid arteries and the proximal segments of the anterior and middle cerebral arteries, triggering compensatory neovascularization at the base of the brain [1]. Despite this adaptive response, the resulting collateral vessels are structurally fragile and hemodynamically insufficient, predisposing affected individuals to both ischemic and hemorrhagic cerebrovascular events [4]. This dual risk contributes to the highly variable clinical presentation of the disease, which ranges from transient ischemic attacks (TIAs) and ischemic stroke to spontaneous intracerebral and intraventricular hemorrhage [5].

The clinical manifestations of MMD differ significantly according to patient age. Pediatric patients most commonly present with ischemic events, whereas adult patients are more frequently affected by hemorrhagic complications, largely attributed to rupture of the fragile collateral vessels [5]. Nevertheless, adult patients may also experience ischemic symptoms, and the coexistence of both hemorrhagic and ischemic events within the same patient's clinical course, although uncommon, has been documented in the literature [3]. Such mixed presentations pose considerable diagnostic and therapeutic challenges, particularly with regard to balancing stroke prevention against the risk of recurrent hemorrhage [6].

Diagnosis relies on a combination of clinical assessment and advanced neuroimaging. While non-contrast computed tomography (CT) and magnetic resonance imaging (MRI) may reveal evidence of prior hemorrhage or infarction, computed tomography angiography (CTA) provides a valuable non-invasive means of identifying characteristic vascular abnormalities [4]. Digital subtraction angiography (DSA) remains the gold standard for definitive diagnosis and staging, enabling precise evaluation of arterial stenosis and collateral vessel formation according to the Suzuki grading system [7]. Early and accurate diagnosis is essential, as timely initiation of appropriate medical or surgical management is critical to preventing recurrent neurological injury [2].

Management of MMD is individualized and may include antiplatelet therapy for ischemic presentations, as well as surgical revascularization procedures such as direct superficial temporal artery to middle cerebral artery (STA-MCA) bypass or indirect techniques including encephaloduroarteriosynangiosis (EDAS) [8,9]. Surgical revascularization has demonstrated efficacy in improving cerebral hemodynamics and reducing long-term stroke risk in appropriately selected patients [7,9]. However, in patients with a concurrent history of intracranial hemorrhage, the decision to initiate antiplatelet therapy remains controversial, requiring careful multidisciplinary evaluation [6,10].

Herein, we report a case of MMD presenting as a TIA in an adult patient with a prior history of recurrent intracerebral and intraventricular hemorrhage. TIA is defined as a transient episode of neurological dysfunction caused by focal brain, spinal cord, or retinal ischemia without evidence of acute infarction, and carries a substantially elevated risk of early ischemic stroke if not promptly recognized and investigated [11]. This case highlights the diagnostic complexity of MMD when ischemic and hemorrhagic manifestations coexist, and emphasizes the critical role of advanced neuroimaging and early neurosurgical referral in optimizing patient outcomes.

## Case presentation

A 45-year-old female was brought to the Emergency Department by Emergency Medical Service with complaints of severe headache, dizziness, and elevated blood pressure. According to the patient, these symptoms had been present for several days; however, their intensity significantly increased during the hours prior to admission, prompting activation of emergency medical services and transport to our clinic. Importantly, the patient reported experiencing transient weakness of the right upper limb accompanied by brief speech disturbance in the period preceding hospital arrival, both of which resolved spontaneously prior to admission.

The patient's past medical history was notable for intracerebral hemorrhage with intraventricular extension two years earlier, managed conservatively and without residual neurological deficits. Additional comorbidities included essential hypertension and iron deficiency anemia. The patient was an active smoker and reported regular use of antihypertensive medications, including Triplixam and moxonidine, as well as Dilox, mirtazapine, and buspirone.

Upon admission, the patient was conscious, alert, and oriented to time and place. Vital signs revealed a blood pressure of 154/90 mmHg, pulse rate of 94 beats per minute, respiratory rate of 18 breaths per minute, temperature of 37.2°C, and oxygen saturation of 97% on room air. General physical examination demonstrated warm, dry skin with preserved turgor and normal mucous membrane coloration.

Neurological examination revealed intact cranial nerve function with round and equal pupils demonstrating preserved bilateral photoreaction. Extraocular movements were conjugate in all directions, facial symmetry was preserved, and the tongue was midline. Motor examination demonstrated a full range of motion with normal strength in all four extremities at the time of assessment. Deep tendon reflexes were moderately brisk and symmetrical bilaterally. Coordination testing was satisfactory on both sides, and the Romberg test was stable. No pathological reflexes or meningeal signs were elicited. Consciousness was assessed using the Glasgow Coma Scale (GCS), yielding a score of E4V5M6, indicative of full consciousness.

Initial laboratory investigations, including complete blood count with differential, serum electrolytes, and infectious disease screening, encompassing hepatitis B surface antigen, hepatitis C antibodies, HIV antibodies, and syphilis serology, were within normal limits. Blood glucose was measured at 103 mg/dL. A 12-lead electrocardiogram demonstrated a regular sinus rhythm without acute abnormalities.

Given the patient's history of prior intracerebral hemorrhage, the transient focal neurological symptoms preceding admission, and the current clinical presentation, CTA of the brain was performed to assess the cerebral vasculature and exclude acute intracranial pathology.

**Figure 1:**
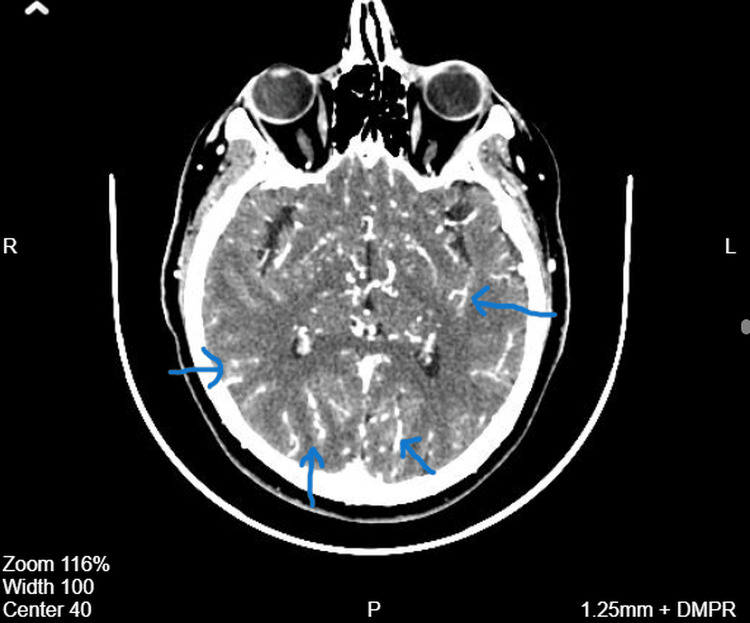
Contrast-enhanced axial CT image demonstrating abnormal collateral vasculature in Moyamoya disease Contrast-enhanced axial computed tomography (CT) image of the brain demonstrating multiple abnormal collateral vessels within the cerebral hemispheres. These tortuous vascular structures represent compensatory collateral circulation characteristic of Moyamoya disease, which develops secondary to progressive stenosis of the distal internal carotid arteries and their proximal branches. Blue arrows indicate the abnormal collateral vessels.

**Figure 2:**
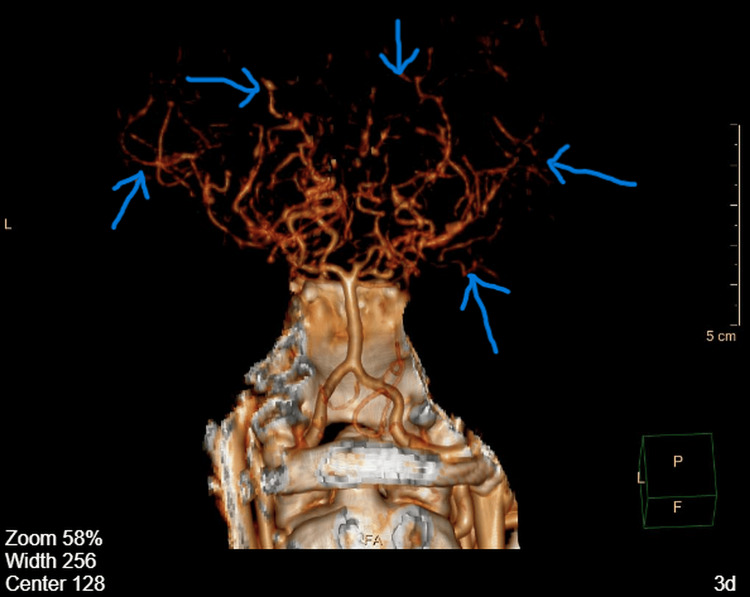
Three-dimensional CT angiography reconstruction demonstrating extensive intracranial collateral circulation in Moyamoya disease Three-dimensional CT angiography (CTA) reconstruction of the intracranial vasculature showing a dense network of abnormal collateral vessels in the bilateral cerebral hemispheres. This abnormal vascular network represents the characteristic collateral circulation seen in Moyamoya disease, formed as a compensatory response to progressive stenosis or occlusion of the intracranial internal carotid arteries and their proximal branches. Blue arrows indicate the abnormal collateral vessels forming the Moyamoya collateral network.

**Figure 3:**
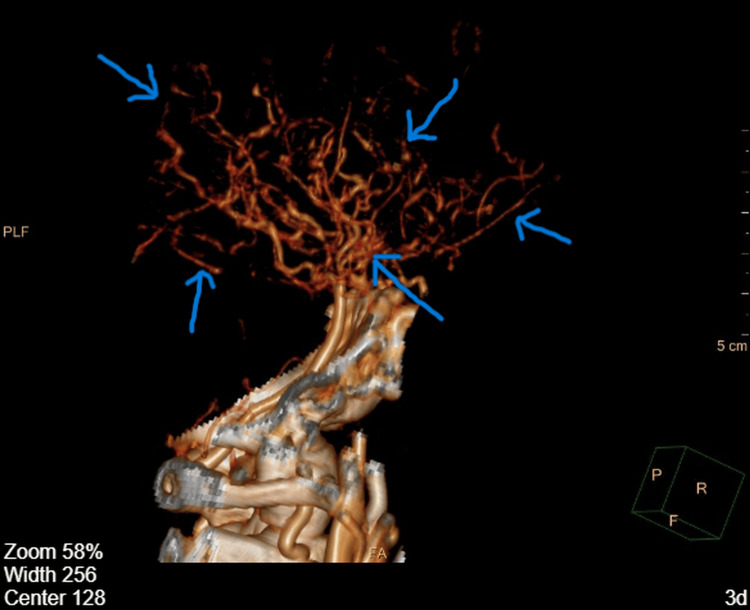
Sagittal three-dimensional CT angiography reconstruction demonstrating collateral vascular network in Moyamoya disease Sagittal three-dimensional CT angiography (CTA) reconstruction of the intracranial vasculature demonstrating abnormal collateral circulation along the lateral aspect of the cerebral hemispheres. These tortuous vascular structures represent compensatory collateral vessels that develop in Moyamoya disease as a response to progressive stenosis or occlusion of the distal internal carotid arteries and their proximal branches. Blue arrows indicate the abnormal collateral vessels forming the Moyamoya collateral network.

The initial contrast-enhanced axial CTA images demonstrated abnormal vascular structures within the anterior cerebral circulation, raising suspicion for an underlying cerebrovascular abnormality (Figure 1). Subsequent coronal CTA reconstruction revealed a reduction of the normal trunk-type arterial pattern within the anterior cerebral circulation, with compensatory collateral vascular networks, while the posterior circulation remained preserved, findings highly suggestive of MMD (Figures 2, 3). The radiologist recommended further vascular evaluation with DSA and neurosurgical consultation. DSA imaging and prior hemorrhagic imaging were not available, representing a limitation of this case.

Additional CT findings demonstrated well-delineated cerebral white matter without focal hyperdense lesions, with no evidence of an acute intracranial process. The ventricular system, basal cisterns, and subarachnoid spaces were symmetrical without evidence of deformation, displacement, or midline shift. Physiologic calcifications were noted in the pineal gland and choroid plexuses. The cerebellar tonsils did not extend below the foramen magnum.

The patient was admitted for dynamic observation. During hospitalization, the general condition remained stable, and the headache gradually subsided. The previously reported transient right upper limb weakness and speech disturbance resolved completely within approximately 24 hours of onset, fulfilling the clinical criteria for a transient ischemic attack in the setting of suspected disease.

The patient was discharged in stable condition with recommendations for outpatient neurological follow-up, neurosurgical consultation, and further vascular evaluation with DSA, as no immediate surgical or endovascular intervention was indicated at the time due to the patient’s stable clinical condition and complete resolution of symptoms.

## Discussion

MMD is a rare, progressive cerebrovascular disorder characterized by stenosis or occlusion of the distal internal carotid arteries and their proximal branches, with resultant formation of abnormal collateral vascular networks at the base of the brain. These fragile collateral vessels produce the pathognomonic angiographic "puff of smoke" appearance from which the disease derives its Japanese name [1]. Although MMD was initially described predominantly in East Asian populations, increasing global case recognition has established that it occurs across diverse geographic regions and ethnic groups, emphasizing the necessity of maintaining a broad index of suspicion in clinical practice [4].

The pathophysiology of MMD centers on the progressive narrowing of major intracranial arteries, most notably the supraclinoid segment of the internal carotid arteries and the proximal portions of the anterior and middle cerebral arteries. In response to advancing arterial stenosis, compensatory neovascularization occurs at the base of the brain in an attempt to sustain adequate cerebral perfusion [6]. However, these newly formed collateral vessels are structurally fragile and hemodynamically inefficient, rendering patients vulnerable to both ischemic and hemorrhagic cerebrovascular events. This dual pathological risk underpins the heterogeneous clinical presentation of MMD and substantially complicates its therapeutic management [8].

The clinical manifestations of MMD are influenced by patient age and disease stage. Pediatric patients predominantly present with ischemic events, including TIAs and ischemic stroke, while adult patients more commonly experience hemorrhagic complications such as intracerebral or intraventricular hemorrhage [3]. Hemorrhagic events in adults are attributed to rupture of the structurally compromised collateral vessels that develop secondary to progressive arterial occlusion. Nonetheless, ischemic manifestations may also occur in adult patients due to progressive cerebral hypoperfusion, reflecting the complex and multifactorial nature of the disease across all age groups [4].

Neuroimaging is indispensable in the diagnosis of MMD. In the acute setting, non-contrast CT serves as the initial modality of choice to exclude intracranial hemorrhage or large territorial infarction; however, dedicated vascular imaging is required to identify the characteristic arterial abnormalities of the disease. CTA has emerged as a valuable non-invasive tool, capable of demonstrating progressive reduction in trunk-type arterial circulation and the presence of abnormal collateral networks [8]. In the present case, CTA revealed atypical anterior circulatory vascular patterns, raising clinical suspicion for MMD and prompting further diagnostic workup. This highlights a key teaching point: the absence of acute findings on non-contrast CT does not exclude significant underlying cerebrovascular pathology [1].

Despite advances in non-invasive vascular imaging, DSA remains the gold standard for definitive diagnosis and staging of MMD. DSA affords superior resolution of intracranial vasculature, enabling precise characterization of the degree of arterial stenosis and the extent of collateral vessel development [5]. The Suzuki grading system, applied through DSA findings, remains essential in guiding both prognosis and treatment planning. Early and accurate diagnosis through advanced neuroimaging is critical, as it enables the timely institution of appropriate management strategies aimed at preventing recurrent cerebrovascular events [1].

The management of MMD poses a substantial therapeutic challenge, particularly in patients presenting with a mixed history of both ischemic and hemorrhagic events. Medical therapy is primarily directed at reducing thrombotic risk and optimizing cerebral perfusion. Antiplatelet agents remain a cornerstone of treatment in patients with ischemic presentations, given their demonstrated efficacy in reducing the incidence of subsequent ischemic stroke [7]. However, the use of antiplatelet therapy carries inherent risks in patients with a prior history of intracranial hemorrhage, as these agents may further compromise the integrity of already fragile collateral vessels and precipitate recurrent hemorrhage. This clinical dilemma, balancing ischemic stroke prevention against hemorrhagic risk, represents one of the most challenging aspects of adult MMD management and demands individualized, multidisciplinary decision-making [9].

For patients in whom medical therapy alone is insufficient, surgical revascularization represents the most definitive and effective long-term treatment strategy. The principal goal of surgical intervention is to restore adequate cerebral perfusion and thereby reduce the cumulative risk of future ischemic or hemorrhagic events. Direct bypass procedures, most notably STA-MCA anastomosis, establish an immediate extracranial-to-intracranial conduit for cerebral blood flow [10]. Indirect revascularization techniques, such as encephaloduroarteriosynangiosis (EDAS), complement direct bypass by facilitating the gradual ingrowth of new collateral vessels over time. A growing body of evidence supports the efficacy of surgical revascularization in improving cerebral hemodynamics and reducing long-term stroke risk in carefully selected patients [5,10].

TIAs represent a clinically significant early sign of MMD and should not be dismissed as benign or self-limiting events. By current tissue-based definition, TIA is characterized by sudden-onset focal neurological dysfunction, most commonly presenting as hemiparesis or speech disturbance in the distribution of a specific vascular territory, without evidence of acute cerebral infarction on neuroimaging [11]. TIA carries a risk of subsequent ischemic stroke of approximately 10-15% within one year of the index event, with the greatest risk concentrated in the first 48 hours, rendering urgent clinical evaluation and vascular imaging mandatory in all affected patients [12]. Clinical risk stratification using validated tools such as the ABCD2 score, which specifically weights motor weakness and speech disturbance as high-risk features, further guides the urgency and intensity of post-TIA workup [13]. Furthermore, large-scale meta-analyses have established that the risk of stroke in the immediate period following a TIA is considerably higher than previously estimated, reinforcing the need for urgent investigation rather than expectant outpatient management [14]. Early initiation of treatment following TIA, including antiplatelet therapy and aggressive vascular risk factor control, has been shown to substantially reduce the risk of subsequent stroke, underscoring the importance of prompt diagnosis and timely intervention [15]. In the present case, the patient experienced transient focal neurological deficits, right upper limb weakness, and brief speech disturbance that resolved within 24 hours, consistent with a TIA [11]. Critically, this ischemic event occurred against a background of prior intracerebral hemorrhage with intraventricular extension, exemplifying the coexistence of both hemorrhagic and ischemic manifestations within a single patient's disease course, a well-recognized but clinically challenging feature of MMD [3].

The present case illustrates several important clinical lessons. First, MMD must be included in the differential diagnosis of patients presenting with transient neurological symptoms, particularly when there is a prior history of unexplained intracranial hemorrhage or recurrent cerebrovascular events, regardless of age, ethnicity, or geographic background [4]. Second, the absence of acute abnormality on initial non-contrast CT should not preclude further vascular investigation, as CTA and ultimately DSA may unmask significant underlying arteriopathy [8]. Third, early neurosurgical referral and advanced vascular imaging are imperative to guide therapeutic decision-making and mitigate the risk of future neurological deterioration [5,10].

In conclusion, this case highlights the complex and bidirectional clinical spectrum of MMD, wherein a single patient may be concurrently at risk for both hemorrhagic and ischemic cerebrovascular events. Early recognition, systematic neurovascular imaging, and coordinated multidisciplinary management are essential pillars in optimizing outcomes and preventing recurrent neurological injury in this vulnerable patient population [2,9].

## Conclusions

MMD is a rare but clinically significant cerebrovascular condition that should be considered in patients presenting with unexplained or mixed ischemic and hemorrhagic cerebrovascular events. This case illustrates that hemorrhagic and ischemic manifestations may coexist during the disease course, creating important diagnostic and therapeutic challenges. The absence of acute abnormalities on initial non-contrast CT should not exclude the possibility of underlying vascular pathology, and further evaluation with advanced neurovascular imaging, including CTA and where indicated DSA, is essential to unmask significant underlying arteriopathy. Early recognition and timely neurosurgical evaluation remain critical for preventing future cerebrovascular complications and improving long-term neurological outcomes.

## Figures and Tables

**Figure 4:**
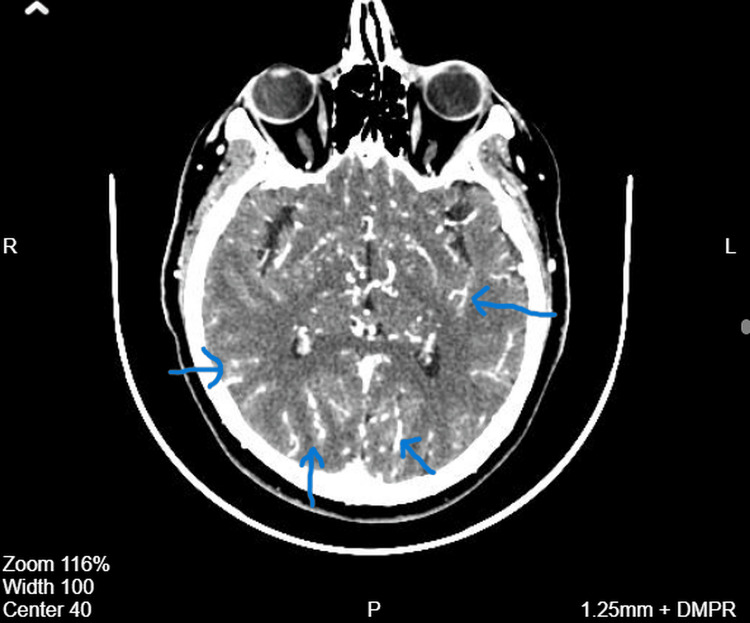
Contrast-enhanced axial CT image demonstrating abnormal collateral vasculature in moyamoya disease Contrast-enhanced axial computed tomography (CT) image of the brain demonstrating multiple abnormal collateral vessels within the cerebral hemispheres. These tortuous vascular structures represent compensatory collateral circulation characteristic of Moyamoya disease, which develops secondary to progressive stenosis of the distal internal carotid arteries and their proximal branches. Blue arrows indicate the abnormal collateral vessels.

**Figure 5:**
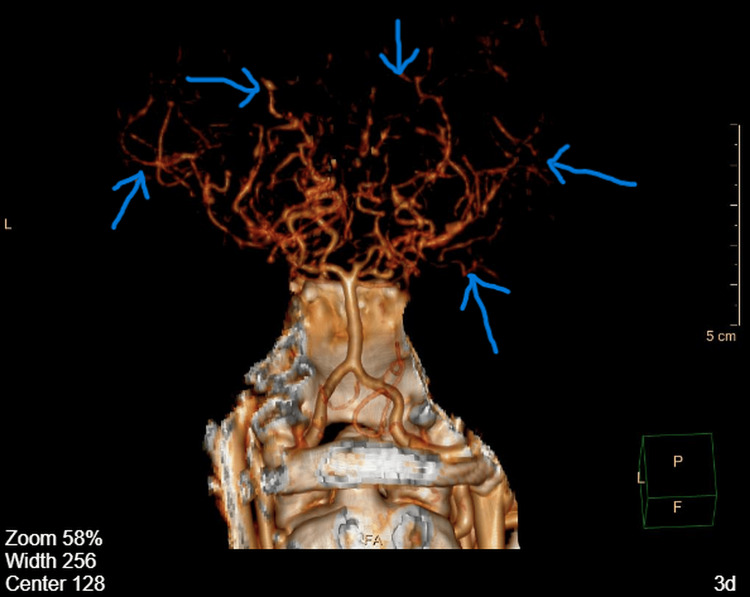
Three-dimensional CT angiography reconstruction demonstrating extensive intracranial collateral circulation in moyamoya disease Three-dimensional CT angiography (CTA) reconstruction of the intracranial vasculature showing a dense network of abnormal collateral vessels in the bilateral cerebral hemispheres. This abnormal vascular network represents the characteristic collateral circulation seen in Moyamoya disease, formed as a compensatory response to progressive stenosis or occlusion of the intracranial internal carotid arteries and their proximal branches. Blue arrows indicate the abnormal collateral vessels forming the moyamoya collateral network.

**Figure 6:**
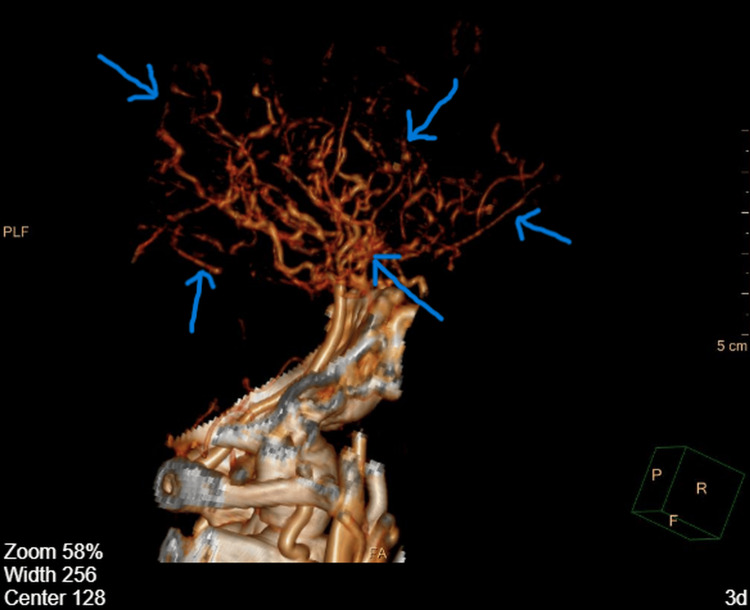
Sagittal three-dimensional CT angiography reconstruction demonstrating collateral vascular network in moyamoya disease Sagittal three-dimensional CT angiography (CTA) reconstruction of the intracranial vasculature demonstrating abnormal collateral circulation along the lateral aspect of the cerebral hemispheres. These tortuous vascular structures represent compensatory collateral vessels that develop in Moyamoya disease as a response to progressive stenosis or occlusion of the distal internal carotid arteries and their proximal branches. Blue arrows indicate the abnormal collateral vessels forming the moyamoya collateral network.
